# TLR4: the fall guy in sepsis?

**DOI:** 10.15698/cst2020.12.237

**Published:** 2020-11-09

**Authors:** Joseph Menassa, Christina Nedeva, Corey Pollock, Hamsa Puthalakath

**Affiliations:** 1 Department of Biochemistry and Genetics, La Trobe Institute of Molecular Science, La Trobe University, Bundoora, Vic 3086, Australia.

**Keywords:** TLR4, TREML4, sepsis, Inflammation, apoptosis

## Abstract

Sepsis and its impact on human health can be traced back to 1000 BC and continues to be a major health burden today. It causes about 11 million deaths world-wide of which, more than a third are due to neonatal sepsis. There is no effective treatment other than fluid resuscitation therapy and antibiotic treatment that leave patients immunosuppressed and vulnerable to nosocomial infections. Added to that, ageing population and the emergence of antibiotic resistant bacteria pose new challenges. Most of the deleterious effects of sepsis are due to the host response to the systemic infection. In the initial phase of infection, hyper activation of the immune system leads to cytokine storm, which could lead to organ failure and this accounts for about 15% of overall deaths. However, the subsequent immune paralysis phase (mostly attributed to apoptotic death of immune cells) accounts for about 85% of all deaths. Past clinical trials (more than 100 in the last 30 years) all targeted the inflammatory phase with little success, predictably, for inflammation is a necessary process to fight infection. In order to identify the regulators of immune cell death during sepsis, we carried out an unbiased, whole genome CRISPR screening in mice and identified Trigger Receptor Expressed in Myeloid-like 4 (Treml4) as the receptor that controls both the inflammatory phase and the immune suppression phase in sepsis (Nedeva *et al.* (2020) Nature Immunol, doi: 10.1038/s41590-020-0789-z). Characterising the *Treml4* gene knockout mice revealed new insights into the relative roles of TLR4 and TREML4 in inducing the inflammatory cytokine storm during sepsis.

## WHOLE GENOME CRISPR SCREEN AND THE IDENTIFICATION OF *Treml4*

**Figure 1:**
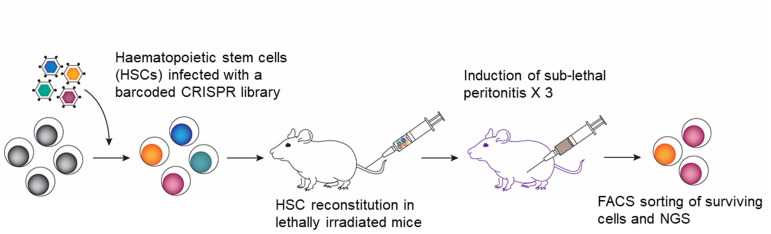
FIGURE 1: The schematic explaining the whole-genome CRISPR screening in mice to identify gene(s) involved in immune cell death during polymicrobial sepsis.

We reasoned that if immune cells undergo apoptosis during sepsis, ablation of the gene responsible for this in a sub-population should give a survival advantage and should predominate the hematopoietic system. We took advantage of the power of the CRISPR editing technology and introduced a library consisting of 95,000 guides representing 25,000 protein coding genes of the mouse genome into mouse hematopoietic stem cells (HSCs; **Figure 1**). We reconstituted lethally irradiated mice with these HSCs and subjected them to three rounds of sublethal peritonitis and the surviving immune cell population was sorted by flow cytometry (based on the BFP fluorescence encoded in the lentiviral backbone of the library vector) followed by DNA sequencing. *Treml4* was identified as the gene enriched about five order of magnitude above the background. It is worth noting that none of the apoptotic genes previously associated with sepsis-mediated lymphopenia such as *Bim* and *Puma* was identified in the screen, suggesting that their effect could be additive and *Treml4* is possibly the upstream regulator of the process.

## PHENOTYPING OF *Treml4*^*−/−*^ MICE

Treml4 protein is a type 1 transmembrane receptor with an extracellular Ig domain and a cytoplasmic DAP12 interaction domain. To understand the function of TREML4 in sepsis, we generated *Treml4*^*-/-*^ mice using the CRISPR/Cas9 system. Upon induction of peritonitis through cecal slurry injection, thymic involution was evident in the wild type (WT) mice, which was absent in *Treml4*^*-/-*^ mice. The thymic atrophy was consistent with thymocyte apoptosis, Bim induction and calcium flux. This is particularly important because thymic involution is one of the hallmarks of neonatal sepsis and the role of the thymus in an individual's immunity is well-documented. For this reason, targeting thymic atrophy has been the subject of recent studies. Thus, thymic atrophy could be used as a surrogate marker for monitoring the effectiveness of therapeutic antibodies in mice with sepsis. However, this was not seen in the peripheral lymphocytes suggesting that additional receptor(s) with a redundant role may be functional in those tissues, which needs further investigation.

Innate immune cells play an important role in the initiation, maintenance and the resolution of the host inflammatory responses. However, sepsis also leads to innate immune cell death, contributing to the risk of secondary infections. Neutrophils account for about 60-70% of white blood cells in humans and are one of the first cells to respond to sites of acute infection and cell damage, playing key roles in the resolution of infections. Neutrophil dysregulation (loss of mature neutrophils i.e. Ly6G^hi^CXCR2^hi^) and impaired bacterial clearance is another feature of sepsis in humans. Consistent with this, significantly less neutrophil dysregulation was seen in *Teml4*^*-/-*^ mice than in WT mice during polymicrobial sepsis, both during the acute inflammatory phase and during the secondary infection phase (*Pseudomonas aeruginosa* lung infection). This was true for other innate immune cells including macrophages and dendritic cells in the blood, bone marrow and in the bronchoalveolar lavage (BAL).

The immunosuppression phase of sepsis is often accompanied by nosocomial infections, particularly *P. aeruginosa.* To study this, we employed a “two-hit” mouse model. *Treml4*^*-/-*^ mice were injected with a sublethal dose of caecal slurry and upon recovery, they were challenged with *P aeruginosa* intra-nasally. Our results showed that there were more functional bronchoalveolar neutrophils in *Treml4*^*-/-*^ mice, which were significantly more capable of phagocytic clearance of *P. aeruginosa* than those from WT littermates, with less myeloperoxidase production and reduced lung damage. Increased clearance of *P. aeruginosa with* reduced myeloperoxidase production in *Treml4*^*-/-*^ mice meant that these mice will be better protected against infection and sepsis compared to the WT littermates. Indeed, *Treml4* deletion afforded almost absolute protection from sepsis.

It is generally believed that lymphocytes play a major role in sepsis pathology. This is based on the observation that sepsis patients exhibit lymphopenia with extensive T/B cell exhaustion. However, in spite of the improved survival of *Treml4*^*-/-*^ mice, lymphocytes from *Treml4*^*-/-*^ and WT mice underwent apoptosis with similar kinetics. Importantly, ablation of neutrophils with anti-Ly6G antibodies reversed the protective phenotype observed in *Treml4*^*-/-*^ mice, underscoring the importance of neutrophils in sepsis. Thus, our results demonstrate that the fast-acting innate immune system is more critical for clearing a fast progressing sepsis infection than the adaptive immune system.

## TLR4 IS NOT THE BOGEYMAN IN INFLAMMATORY SEPSIS

Lipopolysaccharide (LPS) challenge is a widely used model to study leukocyte programming in sepsis with the assumption that TLR4 is the main driver of inflammation. This widely held view was the basis of some of the clinical trials such as Eritonran (Eisai, Japan), which is a TLR4 antagonist, with little success. Another pertinent point is that C3H/HeJ mice with non-functional mutation in TLR4 had normal cytokine response and organ injury indistinguishable from WT mice in response to polymicrobial sepsis. In humans, a non-functional variant of TLR4 (D229G) had no impact on the development or the outcome of polymicrobial sepsis in homozygous and heterozygous individuals. All these observations point to the existence of an alternate receptor that is needed for the cytokine response in sepsis. Our findings with *Treml4*^*-/-*^ mice strongly suggest that TREML4 is the driver of most of the cytokine response mice. Though our *in vitro* studies involving stimulation of TLR4 with LPS and RNASeq analysis revealed that *Treml4* deletion had no impact on cytokine production, LPS administration *in vivo* resulted in subdued cytokine secretion in *Treml4*^*-/-*^ mice for almost all cytokines tested except for IL-6. However, during polymicrobial sepsis, all cytokines tested, including IL-6, were greatly reduced in *Treml4*^*-/-*^ mice. Therefore, we conclude that TREML4 and not TLR4 is the main driver of cytokine storm and *Treml4* deletion offers protection from inflammatory sepsis. However, this reduced cytokine secretion does not seem to have any significant impact on neutrophil migration as significant numbers of functional neutrophils were still observed in the BAL during *P. aeruginosa* infection in *Treml4*^*-/-*^ mice.

## THE SIGNAL TRANSDUCTION PATHWAY IN POLYMICROBIAL SEPSIS

**Figure 2:**
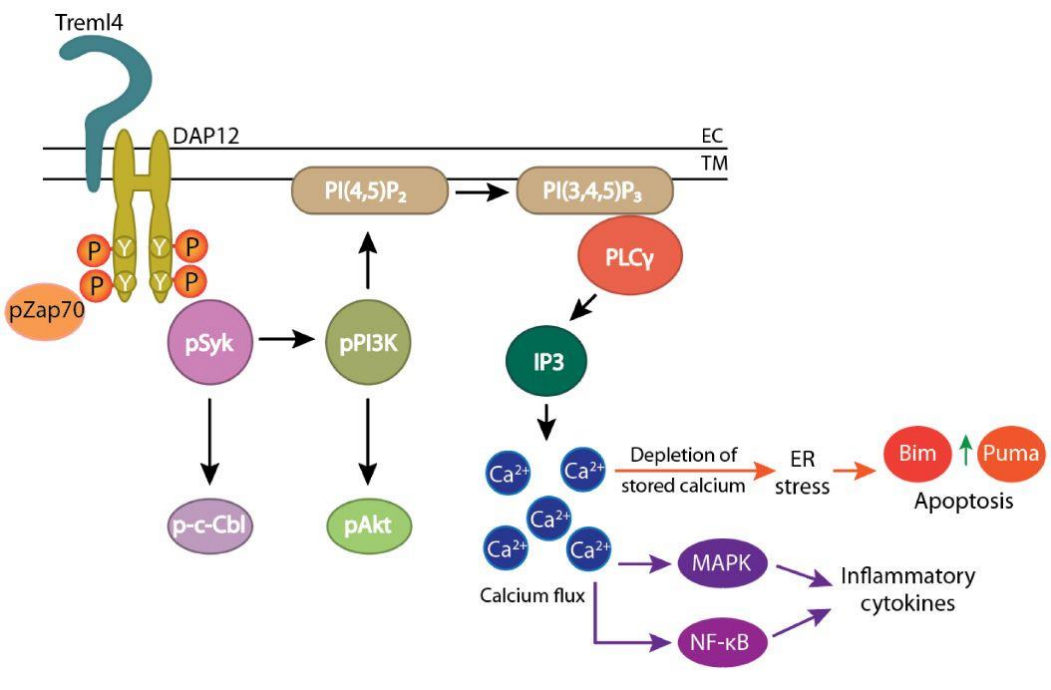
FIGURE 2: TREML4 regulates both the inflammatory phase and the immune suppression phase in polymicrobial sepsis. The schematic depicts the possible players involved.

TREML4 is a TREM family receptor with a cytoplasmic domain capable of engaging the ITAM-bearing molecule DAP-12, involved in signal transduction. DAP-12 signaling is involved in various functions including calcium flux, unfolded protein response (UPR), myeloperoxidase activation and activation of cytokine production through MAPK/ERK or NFκB pathways. Indeed, our biochemical analyses of neutrophils indicate that all these pathways are compromised *Treml4*^*-/-*^ mice, accounting for the phenotype that we observed i.e. reduced cytokine storm and reduced neutrophil apoptosis (**Figure 2**).

Human orthologue of *Treml4* is a pseudogene. However, on chromosome 6, there is group of genes encoding TREM family proteins located and finding the functional equivalent of mouse TREML4 and developing blocking monoclonal antibodies (mAbs) will be the next challenge in developing new therapeutics in treating polymicrobial sepsis.

